# Supplementary material for: Identification of photoperiod-induced specific miRNAs in the adrenal glands of Sunite sheep (Ovis aries)
Source: Front Vet Sci. 2022 Jul 22;9:888207. doi: 10.3389/fvets.2022.888207 (PMC9354845; doi:10.3389/fvets.2022.888207)
Supplement: Supplementary file 1 [file Data_Sheet_1.ZIP › Supplementary file/miRDeep2 file.zip!/miRDeep2 file/Know_Structure/SPLP42A1/oar-mir-432.pdf]

miRBase precursor	: oar-mir-432
Total read count	: 1917
oar-miR-432 read count	: 1912
remaining reads	: 5

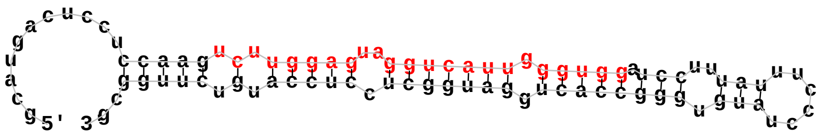

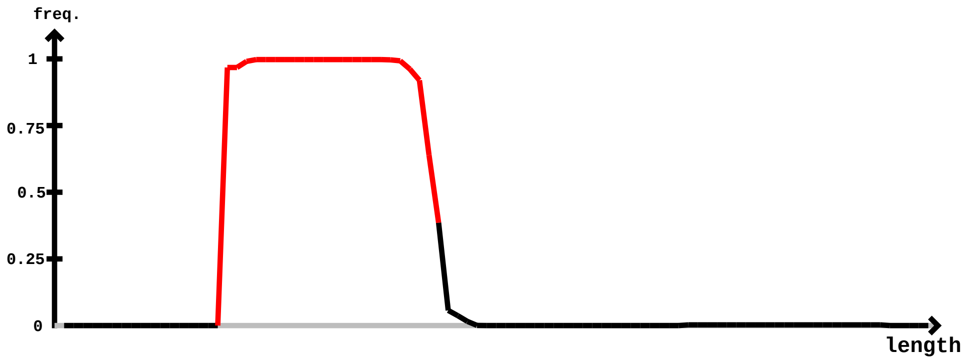


```
                      oar-miR-432
5'- gcaugacuccuccaagucuuggaguaggucauugggugggauccuuuauuucccuaugugggccacuggauggcuccuccaugucuuggcg   -3'    exp
    ..........(((((.(.(((((..(((((((.(((((.(((..(((......))).)))))))).))))))).))))).).)))))..    reads      mm         sample
    ................ucuuggaguaggucauu.........................................................    2          0          seq
    ................ucuuggaguaggucauug........................................................    7          0          seq
    ................ucuuggaguaggucauugg.......................................................    59         0          seq
    ................ucuuggaguaggucauuggg......................................................    81         0          seq
    ................ucuuggaguaggucauugggu.....................................................    537        0          seq
    ................ucuuggaguaggucauugggug....................................................    486        0          seq
    ................ucuuggaguaggucauugggugg...................................................    628        0          seq
    ................ucuuggaguaggucauuggguggga..................................................    37         0          seq
    ................ucuuggaguaggucauuggguggau.................................................    16         0          seq
    ................ucuuggaguaggucauuggguggauc................................................    2          0          seq
    ..................uuggaguaggucauugggug....................................................    1          0          seq
    ..................uuggaguaggucauugggugg...................................................    1          0          seq
    ..................uuggaguaggucauugggugga..................................................    1          0          seq
    ..................uuggaguaggucauuggguggau.................................................    18         0          seq
    ..................uuggaguaggucauuggguggauc................................................    23         0          seq
    ...................uggaguaggucauugggugg...................................................    1          0          seq
    ...................uggaguaggucauuggguggau.................................................    7          0          seq
    ...................uggaguaggucauuggguggauc................................................    4          0          seq
    ...................uggaguaggucauuggguggaucc...............................................    1          0          seq
    ................................................................cuggauggcuccuccaugucu.....    5          0          seq
```
